# Role of Calcium Signaling in GA101-Induced Cell Death in Malignant Human B Cells

**DOI:** 10.3390/cancers11030291

**Published:** 2019-03-01

**Authors:** Simon Latour, Marion Zanese, Valérie Le Morvan, Anne-Marie Vacher, Nelly Menard, Fontanet Bijou, Francoise Durrieu, Pierre Soubeyran, Ariel Savina, Pierre Vacher, Laurence Bresson-Bepoldin

**Affiliations:** 1 Institut Bergonié, Comprehensive Cancer Centre, F-33000 Bordeaux, France; simon_latour@hotmail.fr (S.L.); marionzanese@hotmail.com (M.Z.); V.LeMorvan@bordeaux.unicancer.fr (V.L.M.); anne-marie.vacher@orange.fr (A.-M.V.); ne.menard@laposte.net (N.M.); f.bijou@bordeaux.unicancer.fr (F.B.); f.durrieu@bordeaux.unicancer.fr (F.D.); P.Soubeyran@bordeaux.unicancer.fr (P.S.); 2 Department of Life and Health Sciences, University of Bordeaux, F-33076 Bordeaux, France; 3 INSERM, U1218 ACTION, F-33000 Bordeaux, France; 4 Institut Roche, 92100 Boulogne-Billancourt, France; asavina27@gmail.com

**Keywords:** anti-CD20, GA101, calcium signaling, malignant B cells, cell death

## Abstract

GA101/obinutuzumab is a novel type II anti-CD20 monoclonal antibody (mAb), which is more effective than rituximab (RTX) in preclinical and clinical studies when used in combination with chemotherapy. Ca^2+^ signaling was shown to play a role in RTX-induced cell death. This report concerns the effect of GA101 on Ca^2+^ signaling and its involvement in the direct cell death induced by GA101. We reveal that GA101 triggered an intracellular Ca^2+^ increase by mobilizing intracellular Ca^2+^ stores and activating Orai1-dependent Ca^2+^ influx in non-Hodgkin lymphoma cell lines and primary B-Cell Chronic Lymphocytic Leukemia (B-CLL) cells. According to the cell type, Ca^2+^ was mobilized from two distinct intracellular compartments. In Raji, BL2, and B-CLL cells, GA101 induced a Ca^2+^ release from lysosomes, leading to the subsequent lysosomal membrane permeabilization and cell death. Inhibition of this calcium signaling reduced GA101-induced cell death in these cells. In SU-DHL-4 cells, GA101 mobilized Ca^2+^ from the endoplasmic reticulum (ER). Inhibition of ER replenishment, by blocking Orai1-dependent Ca^2+^ influx, led to an ER stress and unfolded protein response (UPR) which sensitized these cells to GA101-induced cell death. These results revealed the central role of Ca^2+^ signaling in GA101’s action mechanism, which may contribute to designing new rational drug combinations improving its clinical efficacy.

## 1. Introduction

The anti-CD20 monoclonal antibody (mAb), rituximab (RTX), combined with chemotherapy, became the standard regimen for treating non-Hodgkin lymphomas (NHL), such as diffuse large B-cell lymphoma (DLBCL), follicular lymphoma (FL), and chronic lymphocytic leukemia (CLL) [1,2,3]. However, a significant number of patients with DLBCL and most patients with FL or CLL are resistant or relapse [4]. Given that CD20 is highly and specifically expressed on the surface of B cells, it constitutes an ideal therapeutic target in NHL. Therefore, the development of a new anti-CD20 mAb was the subject of intensive research over the past few years.

Currently, anti-CD20 mAbs are generally divided into two distinct classes. Type I mAbs, such as rituximab, induce antibody-dependent cell-mediated cytotoxicity (ADCC), complement dependent cytotoxicity (CDC), and low levels of direct programmed cell death (PCD). Conversely, type II mAbs potently trigger ADCC and PCD, but only weakly elicit CDC [5]. GA101/obinutuzumab is a novel type II anti-CD20 mAb, which was more effective than RTX in preclinical studies, enhancing direct PCD, ADCC, and B-cell depletion in whole blood, as well as antitumor action in human xenograft models [6,7,8]. GA101-induced PCD was shown to involve lysosomal cell death [9] and reactive oxygen species (ROS) production [10]. Clinical studies confirmed the increased efficacy of GA101 versus rituximab monotherapy in CLL; however, best results were obtained using GA101 in combination with chlorambucil or bendamustine [11,12]. In indolent B NHLs, the clinical value of GA101 in monotherapy remains unclear [13]. Thus, a better understanding of its molecular action mechanism to identify pertinent combination regimens to improve therapeutic outcomes is a key challenge. 

Calcium ions (Ca^2+^) are of fundamental importance to signal transduction in animal cells, triggering various cellular processes, such as gene transcription, secretion, cell proliferation, migration, and apoptosis [14]. The intensity and origin of Ca^2+^ signaling determine its specific action. A cytosolic Ca^2+^ rise may originate from intracellular Ca^2+^ stores or extracellular Ca^2+^ influx. In addition to the endoplasmic reticulum (ER), the endolysosomal system is emerging as an important Ca^2+^ storage cell compartment [15,16]. Generally, a rise in cytosolic Ca^2+^ results from the opening of Ca^2+^-permeable channels, expressed either on the plasma membrane or intracellular organelle membranes. Store-operated Ca^2+^ entry (SOCE) is the major extracellular Ca^2+^ influx pathway in non-excitable cells. SOCE is activated by Ca^2+^ release from the ER and involves two key proteins, Orai1 and STIM1 (Stromal interaction molecule 1) [17]. STIM1 acts as an ER Ca^2+^ sensor, while Orai1 represents a channel-forming subunit of the plasma membrane Ca^2+^ release-activated Ca^2+^ (CRAC) channel [18,19]. The inositol 1,4,5 trisphosphate (IP3R) and ryanodine (RyR) receptors, expressed on the ER membrane are the archetypal intracellular Ca^2+^ release channels. More recently, two pore channels (TPC) 1 and 2 were reported to be responsible for Ca^2+^ efflux from lysosomes [20,21]. 

ER Ca^2+^ homeostasis is critical to ER-supported functions, including protein folding and chaperone activity. Its disruption (by Ca^2+^ store depletion) activates ER stress coping responses, including unfolded protein response (UPR) [22]. The ER stress response is mediated by three sensors integrated in the ER membrane: IRE1α (inositol-requiring enzyme 1), ATF6 (activating transcriptor 6), and PERK (protein kinase RNA-like ER kinase), in combination with the ER molecular chaperone immunoglobulin binding protein (BiP) [23]. In the UPR, PERK phosphorylates eukaryotic translation initiation factor (eIF2), which attenuates translation of most messenger RNA (mRNA). One exception is ATF4 (activating transcriptor 4), inducing expression of CHOP (CCAAT-enhancer-binding protein homologous protein) or the pro-apoptotic protein BIM. Upon prolonged, severe activation of the UPR, cells are eliminated by apoptosis [24]. SOCE may indirectly influence ER luminal Ca^2+^-dependent regulation of the UPR, since capacitive Ca^2+^ influx is required to replenish depleted ER Ca^2+^ stores. 

This research investigated the effect of GA101 on Ca^2+^ homeostasis of NHL cell lines and primary B-Cell Chronic Lymphocytic Leukemia (B-CLL) cells with the aim of determining the role of Ca^2+^ signals in GA101-induced cell death. We demonstrated that GA101 triggered a cytosolic Ca^2+^ increase, involving different signaling pathways according to the cell type studied, and that this Ca^2+^ increase played distinct roles in GA101-induced cell death. 

## 2. Results

### 2.1. Calcium Responses Induced by GA101 in NHL Cell Lines

**Figure 1:**
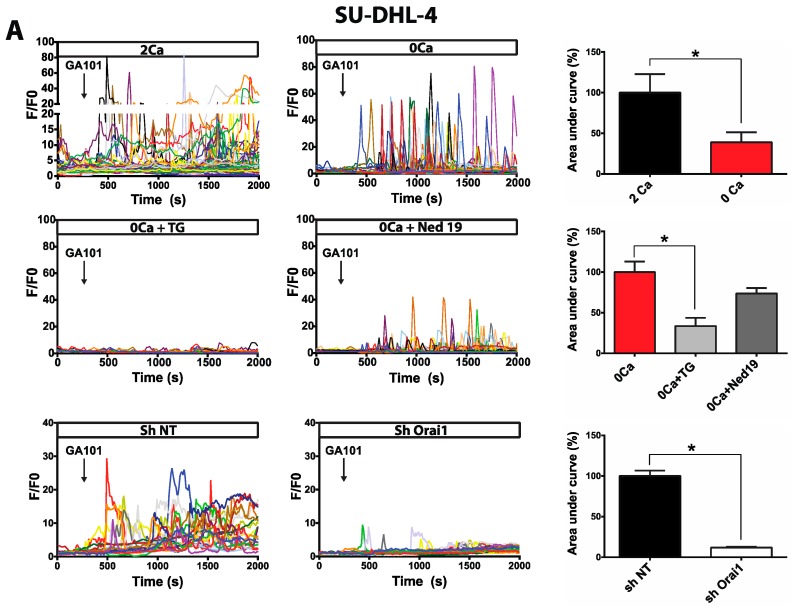

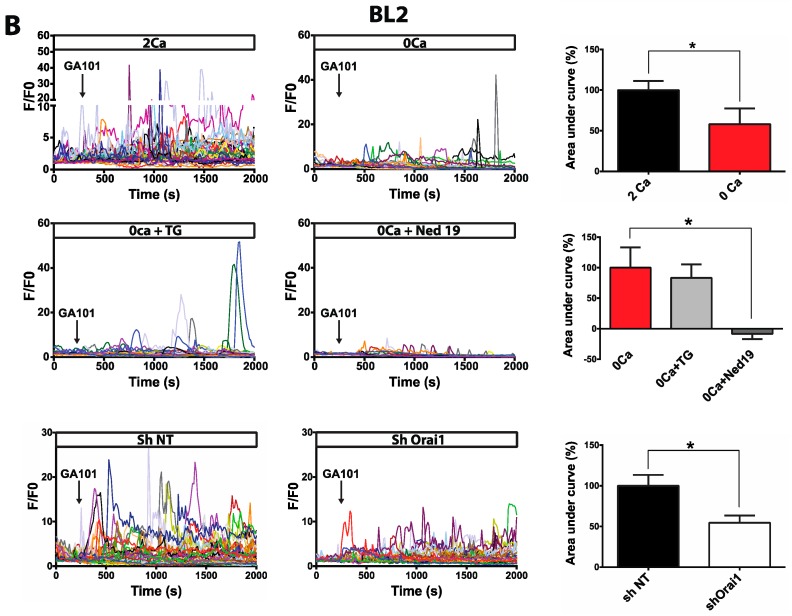
Effect of GA101 on intracellular Ca^2+^ concentration in SU-DHL-4 (**A**) and BL2 (**B**) cell lines. Ca^2+^ responses to GA101 (10 µg/mL) were measured using Fluo2-Leak Resistant-Acetoxy Methyl ester (Fluo2-LR-AM) Ca^2+^ dye and recorded by videomicroscopy (Zeiss LSM 510) using a 25× objective. Black arrows indicate GA101 addition. Each trace represents the response of one cell and data are representative of at least three independent experiments. Data were processed using OriginPro 7.5 (Origin Lab) or GraphPad prism. Cells were recorded in extracellular Hank’s Balanced Salt Solution (HBSS) containing 2 mM Ca^2+^ (2Ca) or in Ca^2+^-free HBSS (0Ca). Cells were preincubated with 100 nM thapsigargin (TG) for 45 min and recorded in Ca^2+^-free HBSS (0Ca + TG) or with 10 µM Ned-19 for 1 h and recorded in Ca^2+^-free HBSS (0Ca + Ned19). Calcium responses to GA101 in cells expressing Non Targeting shRNA (sh NT) or sh Orai1 were recorded in HBSS containing 2 mM Ca^2+^. Histograms represent areas under curves (AUC) calculated, under various recording conditions, between the application time of GA101 and *t* = 2000 s; * *p* < 0.05.

The addition of GA101 triggered a cytosolic Ca^2+^ increase in all cell lines recorded in HBSS (Hank’s Balanced Salt Solution) containing 2 mM Ca^2+^, consisting of multi-peaks and/or a sustained plateau phase, but of lower amplitude and frequency in BL2 and Raji cell lines (Figure 1, Supplemental Figure S1A). To determine the origin of these Ca^2+^ responses, cells were recorded in Ca^2+^-free medium. Ca^2+^ responses were maintained, but the areas under the curves were statistically smaller in all cell types (Figure 1; Figure S1A), suggesting that these responses resulted from both intracellular Ca^2+^ store mobilization and extracellular Ca^2+^ influx. To identify the membrane ion channels involved in the GA101-induced calcium influx, SU-DHL-4 and BL2 cells stably expressing a short hairpin RNA (shRNA) Orai1 were generated (Figure S2). Once again, Ca^2+^ responses induced by GA101 were significantly attenuated in Orai1 knockdown cells compared to cells expressing a non-targeting shRNA (sh NT) (Figure 1). Moreover, cells pretreated with BTP2, a commonly-used SOCE inhibitor, exhibited significantly lower GA101-induced Ca^2+^ responses (Figure S1B). These results revealed that GA101 was able to induce an Orai1-dependent Ca^2+^ influx in NHL-B cells. The next step was to determine the origin of the intracellular Ca^2+^ store mobilization induced by GA101. To this end, cells were pretreated with either thapsigargin (TG, a sarco/endoplasmic reticulum Ca^2+^ ATPase (SERCA) inhibitor, emptying the ER Ca^2+^ pool) or Ned-19 (an inhibitor of two pore channels expressed on lysosome) and recorded in Ca^2+^-free medium. Pretreatment of SU-DHL-4 cells with TG, but not with Ned-19, dramatically decreased the Ca^2+^ response in terms of percentage and amplitude (Figure 1A). In contrast, in BL2 and Raji cells, Ca^2+^ mobilization was unaffected by TG, but abolished by Ned-19 (Figure 1B; Figure S1A). These results revealed that GA101 mobilized Ca^2+^ from ER in SU-DHL-4 cells or lysosomes in BL2 and Raji cells, and subsequently activated Ca^2+^ influx by Orai1-dependent Ca^2+^ channels in all cell lines.

### 2.2. Role of Calcium Influx in GA101-Induced Cell Death

Given that type II anti-CD20 mAbs cause a strong homotypic adhesion leading to cell aggregation, it was suggested by Golay et al. [25] that the analysis of the cell death induced by these Abs using flow cytometry should be interpreted with caution. Other studies clearly showed that cell death could be detected after GA101 treatment by various techniques including flow cytometry [5,26]. In a preliminary approach, we analyzed and compared cell death induced by GA101 by microscopy and flow cytometry after propidium iodide (PI) labeling, two conventional techniques. As shown in Figure S3A, GA101 triggered cell death in all cell lines tested, and the increase in dead cells detected by both methods was of the same order. Thus, regardless of the cell death detection technique used, we observed that BL2 cells were the most sensitive to GA101-induced cell death, while SU-DHL-4 cells were the least. Flow cytometry allowed a rapid analysis of thousands of cells; in the further experiments, cell death was measured using this technique. 

**Figure 2:**
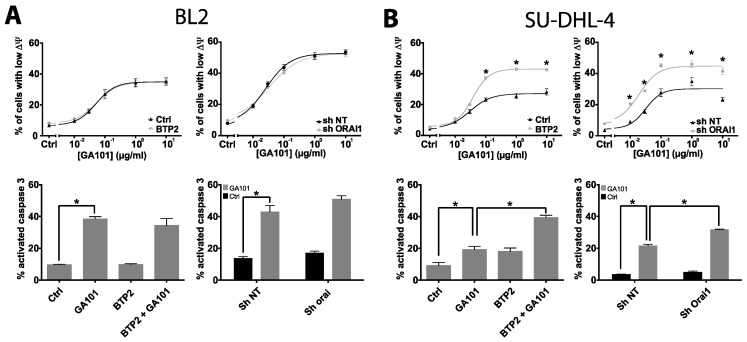
Involvement of store-operated Ca^2+^ entry (SOCE) in GA101-induced cell death. (**A**) BL2 cells. (**B**) SU-DHL-4 cells. Left panels: Cells were incubated with GA101 in the presence or absence of BTP2 (10 µM) for 24 h. Right panels: Cells expressing sh NT or sh Orai1 were treated with GA101 for 24 h. Cell death was assessed by measuring the loss of mitochondrial membrane potential (Δψm), using tetramethylrhodamine methyl ester (TMRM) as a fluorescent dye, or by caspase 3 activation, measured by the FAM-FLICA in vitro caspase detection kit and both analyzed by flow cytometry; * *p* < 0.05.

Orai1-dependent Ca^2+^ influx was reported to exert a negative feedback on RTX-induced apoptosis [27]. Therefore, we examined whether the same type of mechanism was activated by GA101. In BL2 and Raji cells, Orai1 knockdown or BTP2 pretreatment had no effect on GA101-induced cell death (Figure 2A; Figure S3B). In contrast, BTP2 and, to a lesser extent, the downregulation of Orai1 improved the efficacy of GA101 for inducing cell death in SU-DHL-4 cells, (Figure 2B); however, only Orai1 knockdown increased their sensitivity for GA101 (half maximal efficacy concentration (EC_50_) Control = 0.037 ± 0.005 vs. BTP2 = 0.036 ± 0.002 µg/mL, *p* > 0.05; EC_50_ Sh NT = 0.040 ± 0.002 vs. Sh Orai1 = 0.018 ± 0.002 µg/mL, *p* < 0.05) which is likely attributable to the higher specificity of Sh Orai1 than BTP2 to inhibit Ca^2+^ influx. The effects of Orai1 inhibition on GA101-induced cell death in SU-DHL-4 were not due to CD95 engagement since, unlike RTX [27], GA101 was unable to induce CD95 capping formation, a hallmark of CD95 pathway activation (Figure S4).

**Figure 3:**
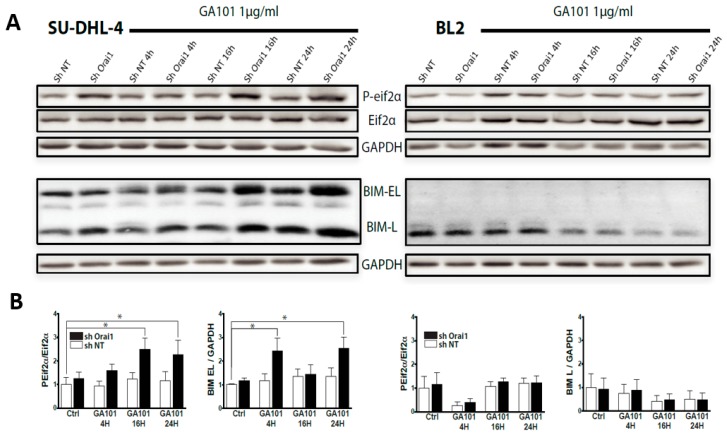
Differential activation of endoplasmic reticulum (ER) stress in SU-DHL-4 and BL2 cell lines. (**A**) Cells expressing sh NT or sh Orai1 were treated with GA101 (1 µg/mL) for varying lengths of time. After lysis, phosphorylated eukaryotic translation initiation factor alpha (P-eIF2α), eIF2α, and BIM expression levels were assessed by immunoblot analysis. GAPDH was used as a loading control. (**B**) Quantification of Western blots is given as means ± SE of three to five independent experiments; * *p* < 0.05.

Disruption of ER Ca^2+^ homeostasis by SERCA inhibition (TG) or Ca^2+^ influx inhibition leads to the accumulation of unfolded proteins and causes ER stress likely to promote cell death [28]. To envisage the involvement of Orai1 inhibition-dependent ER stress in the potentiation of the cell death induced by GA101, we investigated the impact of GA101 on the activation of UPR in cells expressing sh NT or sh Orai1 (SU-DHL-4 and BL2) or after treatment with BTP2 (Raji). To this end, we studied eIF2α phosphorylation and the expression of BIM, one of the targets transcriptionally regulated by CHOP. Our results revealed an increase in eIF2α phosphorylation in under-expressing Orai1 SU-DHL-4 cells treated with GA101. In contrast, no effect of Orai1 under-expression or inhibition was observed in BL2 or Raji cells, respectively (Figure 3; Figure S5A). In agreement with these data, we found that BIM expression increased in SU-DHL-4, while, in BL2 or Raji cells treated with GA101, it decreased with time (Figure 3; Figure S5A). Moreover, we showed that tunicamycin, a main ER stress inducer, sensitized the SU-DHL-4 cell line to GA101-induced cell death but not BL2 or Raji cell lines (Figure S5B). These results reveal a distinctive role of Ca^2+^ entry in GA101-induced cell death, according to the cell line studied. In SU-DHL-4, Ca^2+^ entry repressed cell death by preventing ER stress activation, while, in BL2 and Raji cell lines, Ca^2+^ influx was not, apparently, involved in GA101-induced cell death.

### 2.3. Role of Lysosomes in GA101-Induced Cell Death

**Figure 4:**
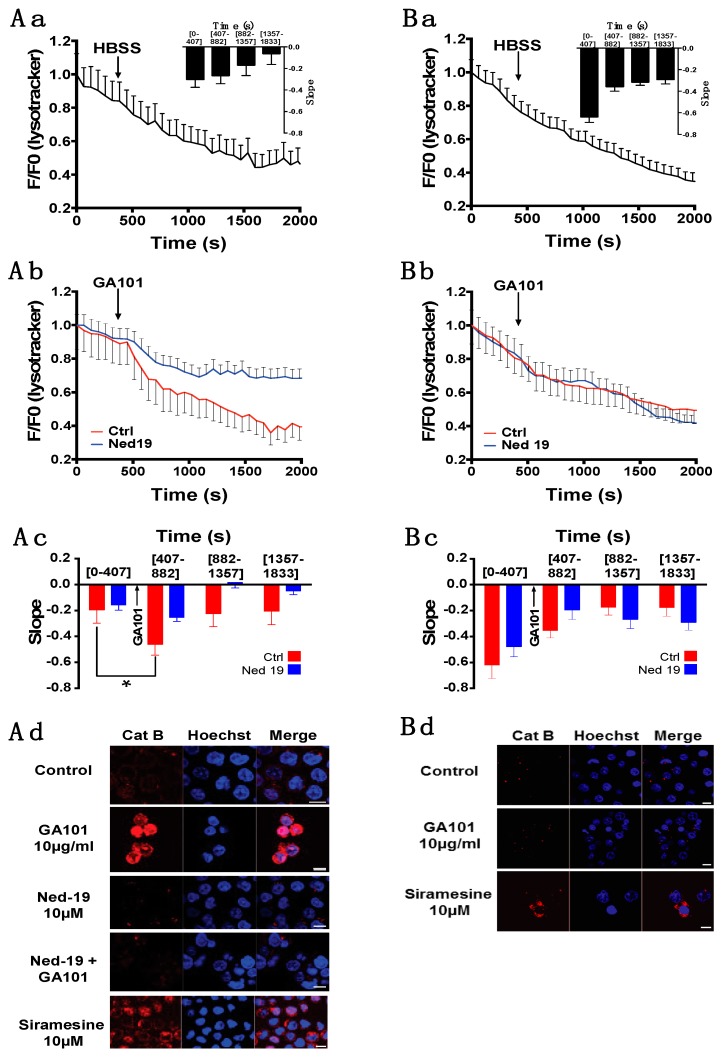

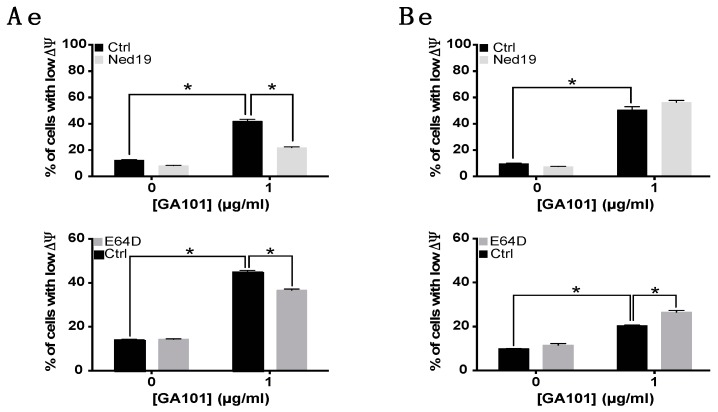
Differential involvement of lysosomal membrane permeabilization (LMP) in (**A**) BL2 cells and (**B**) SU-DHL-4 cells. (**a**) Control recordings of lysotracker fluorescence in HBSS containing 2 mM Ca^2+^. Insert: Lysotracker fluorescence slope quantification over time. (**b**) Effect of GA101 on LMP. The fluorescence of lysotracker red DND-99 was recorded by videomicroscopy (Zeiss LSM 510) using a 63× objective. Black arrows indicate HBSS or GA101 (10 µg/mL) addition. Each trace represents the mean ± SE of three independent experiments. After pretreatment (in blue) or not (in red) with Ned-19 (10 µM) for 45 min, cells were recorded in HBSS containing 2 mM Ca^2+^. (**c**) Quantification of the lysotracker fluorescence slope before and after the addition of GA101 in BL2 and SU-DHL-4. (**d**) Confocal microscopy of cathepsin B staining (red). After treatment, BL2 or SU-DHL-4 cells were fixed and stained with anti-cathepsin B, revealed by donkey anti-goat Ab coupled to Alexa 594. Nuclei were counterstained with Hoechst 33258. Siramesine was used as positive cathepsin B release inducer. Scale bar: 10 µm. (**e**) Effect of LMP induced by GA101 on cell death. Cells were incubated with GA101 in the presence or absence of Ned-19 or E64D for 24 h. Cell death was assessed by measuring the loss of mitochondrial membrane potential (ΔΨm), using TMRM as a fluorescent dye, and then analyzed by flow cytometry; * *p* < 0.05.

Previous works reported the key role of lysosomal membrane permeabilization (LMP) in type II anti-CD20 mAb-induced cell death [9,29,30], and our data suggest that GA101 mobilized lysosomal Ca^2+^ in the BL2 and Raji but not the SU-DHL-4 cell line. To confirm the effect of GA101 on lysosomes, LMP was measured in all cell lines by the decrease in lysotracker fluorescence using videomicroscopy. GA101 provoked a drop in lysotracker fluorescence in BL2 and Raji cells, while no effect was observed in SU-DHL-4 cells or when HBSS was applied to the cell preparation instead of GA101 (Figure 4(Aa,Ab,Ba,Bb); Figure S6A,B). These observations were confirmed after quantification of the fluorescent signal, by calculating the slope of the fluorescence leakage before and after GA101 application. This analysis clearly revealed that GA101 induced a significant increase in the slope ratio in BL2 and Raji but not SU-DHL-4 cells, confirming the activation of LMP in BL2 and Raji but not SU-DHL-4 cells (Figure 4(Ac,Bc); Figure S6C). Moreover, the pre-incubation of BL2 or Raji cells with Ned-19 significantly reversed GA101-induced LMP (Figure 4(Ab,Ac,Bb,Bc); Figure S6B,C). These data suggested that Ca^2+^ efflux from lysosomes by TPC was upstream of LMP and participated in this process. To determine whether the lysosomes released their contents in response to GA101, we performed immunofluorescence staining of cathepsin B, a releasable component of lysosomes. As expected, these experiments confirmed an increase in cathepsin B release in the cytosol of BL2 cells, similar to that observed with the lysosomotropic agent, siramesine, used as a positive control (Figure 4Ad). Note that no cathepsin B release was observed in SU-DHL-4 cells (Figure 4Bd). Pretreatment of BL2 cells with Ned-19 inhibited the GA101-induced cytosolic release of cathepsin B (Figure 4Ad). Finally, we confirmed that LMP was involved in cell death induced by GA101 in BL2 and Raji but not SU-DHL-4 cells, since pretreatment with Ned-19 and E64D, a cathepsin B inhibitor, reduced GA101-induced apoptosis (Figure 4(Ae,Be); Figure S6D). 

In agreement with our calcium-signaling investigations, we revealed that, in BL2 and Raji cells, GA101 activated the release of lysosomal Ca^2+^ leading to the LMP induction and cathepsin B release responsible for cell death. In contrast, in the SU-DHL-4 cell line, GA101 activated a signaling pathway, involving Ca^2+^ influx and ER stress, without the lysosomal pathway playing a major role.

### 2.4. Effect of GA101 on Primary B-CLL

**Figure 5:**
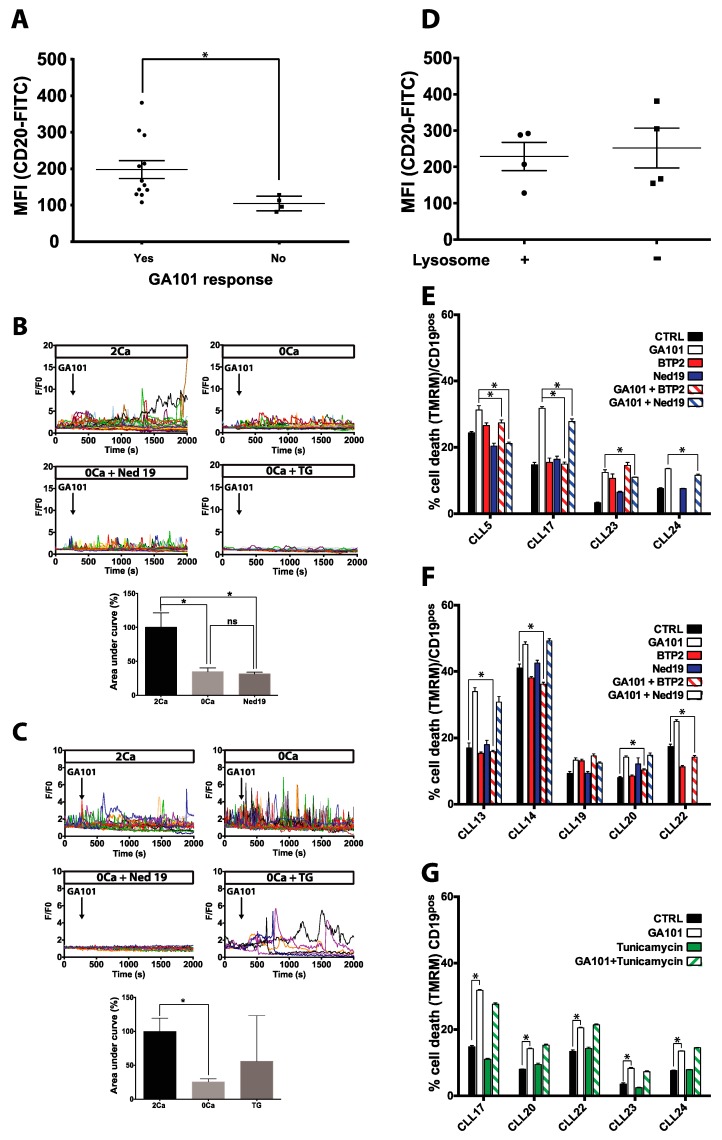
Involvement of Ca^2+^ influx and LMP in GA101-induced cell death in primary B-Cell Chronic Lymphocytic Leukemia (B-CLL). (**A**) Relationship between CD20 expression and the effect of GA101 on cell death in primary human B-CLL. Immediately after isolation peripheral blood mononuclear cells (PBMC) were co-immunostained with anti-CD20-FITC and anti-CD19-PE. The mean fluorescence intensity of CD20 was measured by flow cytometry in CD19-positive (CD19pos) gated cells. PBMCs were incubated with GA101 1 µg/mL for 24 h, and cell death was assessed by measuring the loss of mitochondrial membrane potential (ΔΨm), using TMRM as a fluorescent dye, and analyzed in CD19pos cells by flow cytometry. Cells were considered responders when GA101 induced a statistically significant increase in cell death (Mann–Whitney U; *p* < 0.05 considered as significant). Representative Ca^2+^ responses to GA101 (10 µg/mL) in CLL involving (**B**) TG-sensitive (CLL20) or (**C**) Ned-19-sensitive Ca^2+^ pools (CLL23) recorded in HBSS containing 2 mM Ca^2+^ (2Ca), in Ca^2+^-free extracellular medium (0Ca), after pretreatment with TG (100 nM, 45 min) and recorded in Ca^2+^-free extracellular medium (0Ca + TG), or after pretreatment with Ned-19 (10 µM, 1 h) and recorded in Ca^2+^-free extracellular medium (0Ca + Ned19). Histograms represent areas under curves calculated under various recording conditions, between the application time of GA101 and *t* = 2000 s; * *p* < 0.05. Ca^2+^ responses were recorded as described in Figure 1. (**D**) Relationship between CD20 expression and GA101-induced activation or not of lysosomal Ca^2+^ release in B-CLL. (**E**) B-CLL samples showing lysosome-dependent or (**F**) independent GA101-induced cell death. (**G**) Effect of ER stress inducer on B-CLL cell death. In GA101-responsive B-CLL samples, cells were incubated with GA101 (1 µg/mL) in the presence or absence of tunicamycin 10 ng/mL for 24 h. Cell death was assessed as described above; * *p* < 0.05.

Given that the most encouraging results in clinical trials with GA101 were obtained in patients with B-CLL and that B-CLL is closely related to lymphocytic lymphoma, which is classified as indolent non-Hodgkin’s lymphoma, we tested the effect of GA101 on the death of B-CLL cells harvested from patient blood samples (Table 1). GA101 induced significant cell death in 15 out of 23 B-CLL samples tested (Table 1). The induction of cell death by GA101 is apparently dependent on CD20 expression, since CD20 expression was higher in responsive than non-responsive B-CLL (Figure 5A). We further investigated the effects of GA101 on [Ca^2+^]_i_ of 10 CLLs responsive to GA101-induced cell death. GA101 triggered an intracellular Ca^2+^ increase in all CLLs tested. These Ca^2+^ responses were due to both intracellular Ca^2+^ mobilization and extracellular Ca^2+^ entry (Figure 5B,C). We then determined the origin of GA101-induced Ca^2+^ mobilization. To this end, cells from six B-CLL samples were pretreated with Ned-19 or TG and then recorded in Ca^2+^-free medium. In three CLL samples, Ned-19 completely blocked GA101-induced Ca^2+^ mobilization, while it was unchanged after TG pre-treatment. In contrast, in the three other B-CLL samples tested, Ca^2+^ response was inhibited after TG pretreatment but not after Ned-19 treatment (Figure 5B,C). These data suggested that the Ca^2+^ response to GA101 of some CLL samples was like that of BL2 and Raji cell lines, while others behaved like SU-DHL-4 cells. To decipher the molecular mechanisms responsible for the activation of lysosomal Ca^2+^ release or not in cells, we investigated the expression of CD20, CD38, TPC1, and TPC2 proteins. However, no difference was observed between GA101-induced lysosomal Ca^2+^ release cells or not (Figure 5D; Figure S7). 

The next step was to investigate the involvement of Ca^2+^ signaling in GA101-induced cell death in B-CLL samples. Due to the toxicity of pre-treatments on some B-CLL samples, the effects of BTP2 and Ned-19 on GA101-induced cell death were analyzed in nine B-CLL samples. In one out of nine B-CLL samples, no treatment had any effect on GA101-induced cell death (CLL 19). In four CLL samples (CLL5, CLL17, CLL23, and CLL24), Ned-19 statistically inhibited cell death (Figure 5E). Finally, we observed that BTP2 had no effect (CLL 19 and CLL 23) or inhibited GA101-induced cell death (CLL17, CLL20, CLL14, CLL13, CLL5 and CLL22) (Figure 5E,F). We hypothesized that this latter result may be due to B-CLL resistance to ER stress. Thus, we tested the effects of tunicamycin on basal and GA101-induced cell death in five B-CLL samples. Our results revealed that tunicamycin did not induce cell death in B-CLL or sensitize cells to GA101 (Figure 5G), suggesting that CLL may be resistant to ER stress.

## 3. Discussion

The role of Ca^2+^ in cell death was extensively investigated [31]; however, its pro- or anti-apoptotic effects seem highly dependent on the stimulus and cell type, as well as the Ca^2+^ toolkit that enables cells to specifically regulate Ca^2+^-dependent cell death. Here, we investigated the effect of GA101 on Ca^2+^ signaling and its involvement in the direct cell death evoked by GA101. We identified some key findings: (1) GA101 triggered an intracellular Ca^2+^ increase by mobilizing intracellular Ca^2+^ stores and activating Orai1-dependent Ca^2+^ influx in the three studied NHL cell lines; (2) lysosomal Ca^2+^ release and subsequent LMP were responsible for the cell death induced by GA101 in BL2 and Raji but not in SU-DHL-4 cells; (3) in SU-DHL-4 cells, cell death was repressed by Orai1-dependent Ca^2+^ influx and, consequently, enhanced by Orai1 inhibitors through the activation of UPR in response to ER stress; (4) in B-CLL, GA101 triggered an intracellular Ca^2+^ increase involving mobilization of either lysosomal or ER Ca^2+^ pools and a Ca^2+^ influx, which are differentially implicated in GA101-induced cell death. Taken together, these results revealed the central role of GA101-induced Ca^2+^ signaling in its action mechanism.

GA101 was found to activate two distinct Ca^2+^ responses: one similar to that described with RTX [27], involving ER Ca^2+^ store mobilization and capacitive Ca^2+^ influx in SU-DHL-4 cells, and another involving lysosomal Ca^2+^ release and extracellular Ca^2+^ influx, in BL2 and Raji cell lines. Lysosomes express Ca^2+^ channels, including TPC1 and TPC2, which are activated by NAADP (Nicotinic acid adenine dinucleotide phosphate) [32,33,34]. In BL2 and Raji cells, Ned-19, a NAADP receptor antagonist, was found to suppress Ca^2+^ mobilization triggered by GA101, suggesting that GA101 induces NAADP formation and TPC-dependent Ca^2+^ release from lysosome. Moreover, this lysosomal Ca^2+^ release is probably involved in LMP, since LMP was significantly inhibited by pre-treatment with Ned-19. Interestingly, these two Ca^2+^ signaling pathways were also observed in B-CLL with a ratio of 50/50. In order to identify which molecular mechanism determined the type of Ca^2+^ responses activated by GA101 in cells, we evaluated the expression of various proteins in NHLB cell lines and B-CLL. Unlike for the induction of cell death by GA101 which is related to CD20 expression level, no difference in CD20 expression was observed between lysosomal responsive cells or not. Another explanation could be that CD38 expression, which is responsible for the transformation of NAD+ (Nicotinamide adenine dinucleotide) to the active second messengers NAADP and cADPr (cyclic ADP ribose) [35], may be differentially expressed in B-NHL and B-CLL cells, constituting a switch between the two GA101-activated signaling pathways; however, this hypothesis was not confirmed by our results obtained in cell lines and tested B-CLL. Similarly, TPC1 and TPC2 expression was unchanged between cells. However, these data should be confirmed in a larger cohort of B-CLL. Finally, we cannot exclude that the different Ca^2+^ responses induced by GA101 are due to the origin of the NHL (DLBCL or Burkitt lymphoma) or B-CLL. Further experiments will be necessary to identify the molecular process responsible for activating or not LMP pathway in these cells.

We and others [36,37] showed that RTX-induced Ca^2+^ response in NHLB was due to IP3-dependent intracellular Ca^2+^ mobilization and Orai1-dependent Ca^2+^ influx, while we demonstrated that GA101 was able to trigger at least two different molecular pathways for Ca^2+^ signaling according to the cell type, which could contribute to its better clinical efficacy than RTX. Indeed, type I CD20 antibody RTX and type II CD20 antibody GA101 differ fundamentally in their interaction with CD20, which may account for activation of various transduction pathways, including Ca^2+^ signal kinetics and/or more or less antibody efficiency [38]. 

In human primary B-CLL samples, we observed heterogeneity in cell death responses induced by GA101, which may be due to individual variability in CD20 expression or to BCR (B-cell receptor) functional signaling and/or to IGHV (immunoglobulin heavy-chain variable region) mutational status. Indeed, CLL IGHV mutational status reveals bifurcation of responses toward proliferation or anergy [39], and one can hypothesize that anergic mutated IGHV CLL cells could be more sensitive to GA101-induced cell death than proliferative unmutated IGHV CLL because their increased susceptibility to apoptosis. Further experiments should be necessary to elucidate this point. 

It was previously shown that GA101 triggered LMP and the subsequent cathepsin release responsible for protein cleavage (including caspases) and cell death [9,40]. We confirmed this mechanism and suggest that LMP and cell death are, at least partly, initiated by lysosomal Ca^2+^ release, since Ned-19 inhibited LMP and GA101-induced cell death in BL2, Raji cells, and in half of B-CLL samples. In contrast, in the SU-DHL-4 cell line, GA101 was unable to trigger LMP. Based on the slope of lysotracker fluorescence in resting conditions (before GA101 application) being higher in SU-DHL-4 than those observed in BL2 and Raji cells, it could be possible that LMP could not be observed in these cells. However, this absence of LMP is correlated with the lack of lysosomal Ca^2+^ mobilization, with the absence of cathepsin release in response to GA101 and with no effect of Ned-19 or E64D on cell death induced by GA101 suggesting that lysosomal cell death is not activated by GA101 in these cells. In SU-DHL-4 cells, Orai1-dependent Ca^2+^ influx was clearly shown to repress sensitivity to GA101-induced cell death, since pharmacological or genetic inhibition of Orai1 activity improved the effect of GA101. This repressive effect of the Orai1-dependent Ca^2+^ influx on GA101-induced cell death is similar to that observed with RTX; however, in contrast to RTX, this effect was not attributable to Ca^2+^ inhibition of CD95 engagement [27,41], but rather the impediment of ER stress. Indeed, Orai1 inhibition increased the phosphorylation level of eIF2α, as well as the expression of BIM, two ER-stress markers [28]. In the SU-DHL-4 cell line, GA101 triggered ER Ca^2+^ store release, but activated capacitive Ca^2+^ influx, thus refilling the Ca^2+^ stores and preserving Ca^2+^ homeostasis and the efficiency of ER. When this Ca^2+^ influx was inhibited, the reticular Ca^2+^ concentration remained low, inducing accumulation of unfolded proteins, causing ER stress and BIM expression to increase, which promoted cell death [28,42] In BL2, Raji, and B-CLL cells, although GA101 triggered a Ca^2+^ influx, its inhibition did not sensitize cells to GA101-induced cell death. Two hypotheses may explain this result. The first is that BL2, Raji, and B-CLL cells are resistant to ER stress, as suggested by experiments where ER-stress inducers were unable to induce cell death or potentiate GA101-induced cell death. This resistance to ER stress may be due to Bcl-2 overexpression, as already described in B-CLL [43,44], which, by interacting with IP3R [45], may repress ER Ca^2+^ release and, in turn, protect cells from ER stress. The second possibility is that Orai1-dependent Ca^2+^ entry is not the main source of Ca^2+^ influx in these cells, but other Ca^2+^ channels, such as TRPC (Transient Receptor Potential channels Canonical) [46] or TRPV (Transient Receptor Potential channels Vanilloid) [47] channels, may be significant contributors to Ca^2+^ entry. 

## 4. Materials and Methods

### 4.1. Reagents and Antibodies

GA101 was kindly provided by Roche Glycart AG. Thapsigargin and tunicamycin were purchased from Merck Millipore (Fontenay s/s bois, France). BTP2, Ned-19 trans, and puromycin were supplied by Tocris Bioscience (Lille, France). Hoechst 33258 and siramesine were purchased from Sigma-Aldrich (L’Isle d’Abeau, France). Tetramethylrhodamine methyl ester (TMRM) and lysotracker red DND-99 were from ThermoFisher Scientific (Courtaboeuf, France), and Fluo2-leak resistant (LR)- acetoxymethyl ester (AM) was from Euromedex (Mundolsheim, France). FAM-FLICA in vitro caspase 3 detection kits were supplied by AbD Serotec (Kidlington, UK). 

Anti-human Orai1 rabbit polyclonal antibody was from Alomone Labs (Jerusalem, Israel). The anti-human CD19-PE, CD19-488 (clone HIB19), and anti-human CD20-FITC (clone 2H7) and their respective isotype controls were provided by eBiosciences (San Diego, CA, USA). Anti-cathepsin B and anti-BIM were supplied by Santa Cruz Biotechnology (Heidelberg, Germany). The anti-phospho eIF2α and anti-eIF2α were from Cell Signaling Technology (Ozyme, France). Alexa 594-conjugated donkey anti-goat came from Life Technologies (Saint Aubin, France). Horseradish peroxidase (HRP)-conjugated goat anti-mouse and goat anti-rabbit were from ThermoFisher Scientific.

### 4.2. Patient Samples and Cell Lines

Patients were recruited under the Institut Bergonié Institutional Review Board approval (n°2013-H-001, date 12-02-2013) and informed consent process, in accordance with the Declaration of Helsinki. For B-cell chronic lymphocytic leukemia (B-CLL), peripheral blood mononuclear cells (PBMCs) were isolated from whole blood by Ficoll gradient centrifugation, washed and cultured in RPMI 1640 (Life Technologies) supplemented with 10% FBS (Fetal Bovine Serum). All B-CLL samples were tested for cell death in response to GA101. The other tests (Ca^2+^ measurements and/or pharmacological studies) were performed on GA101-responsive B-CLL according to the quantity of biological material available. For Ca^2+^ measurement and flow cytometry experiments, B cells were identified after staining with fluorescent anti-CD19 antibody. The BL2 and Raji Burkitt lymphomas and SU-DHL-4 FL-transformed cell lines were obtained from the DMSZ (Deutsche Sammlung von Mikroorganismen und Zellkulturen) cell collection (Braunschweig, Germany).

### 4.3. Short Hairpin RNA Lentivirus Transduction

The short hairpin RNA (shRNA) lentivirus transduction approach was used to knockdown orai1 gene expression. pLKO1 lentiviral vectors expressing DNA sequences encoding for Orai1 shRNA (TRCN0000165044 and TRCN0000161221) were purchased from Sigma-Aldrich. Non-targeting shRNA (sh NT) was used as a lentivirus cell transduction control. Lentiviruses were produced as previously described [27]. The titer of each lentiviral batch was determined on various cell lines. Transduced cells were selected by puromycin treatment.

### 4.4. Apoptosis Assays

B-cells were untreated or pretreated with various pharmacological agents for 1 h and then incubated with or without GA101 for 24 h. Apoptosis was detected by the loss of mitochondrial membrane potential using TMRM as a fluorescent dye. TMRM is a fluorescent lipophilic cation which accumulates in hyperpolarized mitochondria of living cells, but not in depolarized mitochondria of apoptotic cells [48]. Thus, after treatment, cells were incubated with 200 nM TMRM for 20 min, and the loss of cell fluorescence, corresponding to apoptotic cells, was measured by flow cytometry. Apoptosis was also assessed by measuring the percentage of cells with active caspase 3, detected by the FAM-FLICA in vitro caspase detection kit, used according to the manufacturer’s instructions. Apoptotic cells were analyzed by flow cytometry using a FacsCalibur cytometer and Cell Quest software (BD biosciences, Le Pont de Claix, France). 

### 4.5. Intracellular Calcium Measurement

For single-cell [Ca^2+^]_i_ measurement cells were loaded with 7.5 µM Fluo2- LR-AM (λex: 488 nm, λem 515 nm) calcium dye, which exhibits limited compartmentalization in intracellular stores and is leakage resistant, in the presence of 0.02% pluronic F127 in HBSS at room temperature for 25 min. The cells were rinsed with HBSS and incubated without Ca^2+^ probe for 15 min to complete de-esterification of the dye. For B-CLL, cells were previously labeled with anti-human CD19 APC (Allophycocyanin) at room temperature for 20 min, to ensure that Ca^2+^ recordings originated from B cells. In some experiments, cells were placed in a Ca^2+^-free HBSS medium, to which 100 µM EGTA (ethylene glycol-bis(β-aminoethyl ether)-*N*,*N*,*N*′,*N*′-tetraacetic acid) was added to chelate any residual Ca^2+^ ions. This medium was added to the cells just before recording to avoid leaks from the intracellular calcium stores.

Fluorescence was recorded using an inverted fluorescence confocal microscope (Zeiss LSM 510, Göttingen, Germany) with a 25× oil immersion objective. Images were recorded at constant 10-s intervals. Regions of interest corresponding to cells recorded were drawn to analyze the fluorescence signal. To overcome putative differences of Ca^2+^ probe concentration between cells, we normalized the fluorescence signal (F) of each cell by its starting basal fluorescence (F0). Data were processed using OriginPro 7.5 (Origin Lab, Paris, France) and Prism 6.0 (Graphpad, San Diego, CA, USA) software. 

### 4.6. Assessement of Lysosomal Membrane Permeabilization

Lysosomal membrane permeabilization (LMP) and lysosomal cathepsin B release were assessed by lysotracker staining and immunofluorescence, respectively, and analyzed by fluorescence microscopy. For LMP detection, cells were stained with lysotracker red (100 nM) in HBSS at 37 °C for 45 min. Cells were then rinsed twice in HBSS. Fluorescence micrograph images were obtained using an inverted fluorescence microscope with an ApoPLAN 63× objective. Images were recorded at constant 1-min intervals. Regions of interest corresponding to cells recorded were drawn to analyze the fluorescence signal, and its slope was calculated using GraphPad Prism software. 

To visualize cathepsin B release in the cytosol, cells were untreated or treated with GA101 or siramesine (a lysosomotropic agent), and immunofluorescence was performed. Cells were fixed in Phosphate Buffer Saline (PBS) containing 4% *w*/*v* paraformaldehyde at 4 °C for 10 min and then permeabilized in PBS supplemented with 5% Bovine Serum Albumin (BSA)/0.1% saponin for 5 min. Cells were incubated with anti-cathepsin B in PBS/1% *w*/*v* BSA at 4 °C overnight. Cathepsin B was revealed using secondary Alexa594-coupled donkey anti-goat Ab. Nuclei were stained using Hoechst 33258. Images were acquired using a Zeiss LSM 510 meta confocal microscope with an ApoPLAN 63× objective.

### 4.7. Western Blotting

Cell lysates were prepared using a lysis buffer containing protease and phosphatase inhibitors (Cell Signaling Technology). The lysates were separated by SDS-PAGE and transferred to Polyvinylidene fluoride (PVDF) membrane using an iBlot Gel Transfer System (Thermofisher Scientific). The membranes were incubated with 5% nonfat milk w/v in PBS buffer for 1 h and then reacted with the primary Ab in PBS/Tween buffer with 5% nonfat milk w/v by shaking at 4 °C overnight. The appropriate second Abs conjugated to HRP was used to detect the protein of interest via Enhanced Chemiluminescence (ECL) (Millipore). 

### 4.8. RT-qPCR

Total RNA was isolated using the RNeasy kit (Qiagen, Les Ullis, France) and its concentration was determined by Nanodrop. Quality and integrity of total RNA was assessed using the Bioanalyzer (Agilent, Les Ullis, France). For real-time PCR, the reverse transcription (RT) reaction was carried out with 1 µg of total RNA in a 20-µL mixture to generate first-strand complementary DNA (cDNA) using PrimeScript RT reagent kit with genomic DNA (gDNA) Eraser (Takara, Nice, France). All real-time PCR reactions were performed in a 20-µL mixture containing 1/2 volume of cDNA preparation using PowerUp Sybr Green Master mix (Thermofischer Scientific) and Taq Ozyme HS (Ozyme) according to manufacturer’s protocol. Quantitative PCR was performed using appropriate primers (CD38 forward: TCA-GCC-ACT-AAT-GAA-GTT-GGG-A; CD38 reverse: CTG-GAC-CTG-TGT-GAA-CTG-ATG-G; TPC1 forward: GGA-GCC-CTT-CTA-TTT-CAT-CGT; TPC1 reverse: GGA-GCC-CTT-CTA-TTT-CAT-CGT; TPC2 forward: GTA-CCC-CTC-TTG-TGT-GGA-CG; TPC2 reverse: GGC-CCT-GAC-AGT-GAC-AAC-TT). Real-time quantifications were performed using the StepOnePlus Real-Time PCR System (Applied Biosystems, Thermofisher Scientific). The fluorescence threshold value was calculated using StepOne software (Applied Biosystems).

### 4.9. Statistical Analysis

All data were expressed as means ± SE. The significance of differences was calculated using the parametric *t*-test or one-way ANOVA test or the non-parametric Mann–Whitney U or Wilcoxon tests, as appropriate (* *p* < 0.05).

## 5. Conclusions

In conclusion, this study provides new mechanistic insights into GA101-induced cell death, including the important, complex role of Ca^2+^ signaling. The source of Ca^2+^ increase, as well as its role in GA101-induced cell death, differed according to the cell type studied. Further investigations are required to identify the biomarkers of Ca^2+^ involvement in GA101-induced cell death, in order to design new, rational drug combinations to improve the clinical efficacy of GA101.

## Figures and Tables

**Table 1:** B-CLL patient information; GA101 1µg/mL for 24 h; * *p* < 0.05.

Clinical Characteristics						GA101-Induced Cell Death in CD19^pos^ Cells (% of Control)
Patient ID	Age/Gender	Stage	TP53 (del17p)	% WBC	% CD19^pos^	
**CLL1**	79/F	A	Positive	78	91.2	9.7 *
**CLL2**	73/F	C	Positive	69	82.1	2
**CLL3**	77/F	A	Negative	55	82	−15.7
**CLL4**	60/F	A	Negative	35	66	22 *
**CLL5**	79/F	C	Negative	56	76.8	6.9 *
**CLL6**	75/M	A	Negative	24	56.7	8.2 *
**CLL7**	79/F	A	Negative	45	23	0.2
**CLL8**	66/F	A	Negative	34	50.4	5.2 *
**CLL10**	62/F	A	Negative	39	66	−2
**CLL11**	86/F	A	Negative	86	92	1.8
**CLL12**	70/F	A	Negative	33	68	10.7 *
**CLL13**	66/F	A	Negative	36	66	15.8 *
**CLL14**	73/F	A	Negative	38	63	7 *
**CLL15**	56/M	A	Negative	23	51	−1.5
**CLL16**	67/M	A	Negative	58	76	1
**CLL17**	43/F	A	Negative	36	69	16.9 *
**CLL18**	73/F	A	Negative	41	81	−1.2
**CLL19**	54/M	A	Negative	55	73	4 *
**CLL20**	51/F	A	Negative	24	47	6.2 *
**CLL21**	74/F	B	Postive	69	92	6.8 *
**CLL22**	70/M	A	Negative	69	85	7.5 *
**CLL23**	49/M	B	Negative	52	87	9.1 *
**CLL24**	75/M	B	Negative	62	86	5.9 *

F: female, M: male, CLL: Chronic lymphocytic leukemia, WBC: White blood cells.

## Supplementary Materials

The following are available online at https://www.mdpi.com/2072-6694/11/3/291/s1: Figure S1: Effect of GA101 on intracellular Ca^2+^ concentration in the Raji cell line, Figure S2: Validation of SU-DHL-4 and BL2 cell lines infected with non-targeting or Orai1-targeting shRNA lentiviruses, Figure S3: Cell death induced by GA101 in various cell lines measured by microscopy or flow cytometry, Figure S4: Unlike rituximab, GA101 is unable to induce CD95 capping, Figure S5: Involvement of ER stress in GA101-induced cell death, Figure S6: Effect of GA101 on LMP in the Raji cell line, Figure S7: Messenger RNA expression level of CD38, TPC1, and TPC2 in NHL cell lines and B-CLLs.
