# Supplementary material for: Role of Calcium Signaling in GA101-Induced Cell Death in Malignant Human B Cells
Source: Cancers (Basel). 2019 Mar 1;11(3):291. doi: 10.3390/cancers11030291 (PMC6468563; doi:10.3390/cancers11030291)
Supplement: Supplementary file 1 [file cancers-11-00291-s001.pdf]

Supplementary Materials: Role of Calcium Signaling in GA101-Induced Cell Death in Malignant Human B Cells

Simon Latour, Marion Zanese, Valérie Le Morvan, Anne-Marie Vacher, Nelly Menard, Fontanet Bijou, Francoise Durrieu, Pierre Soubeyran, Ariel Savina, Pierre Vacher and Laurence Bresson-Bepoldin

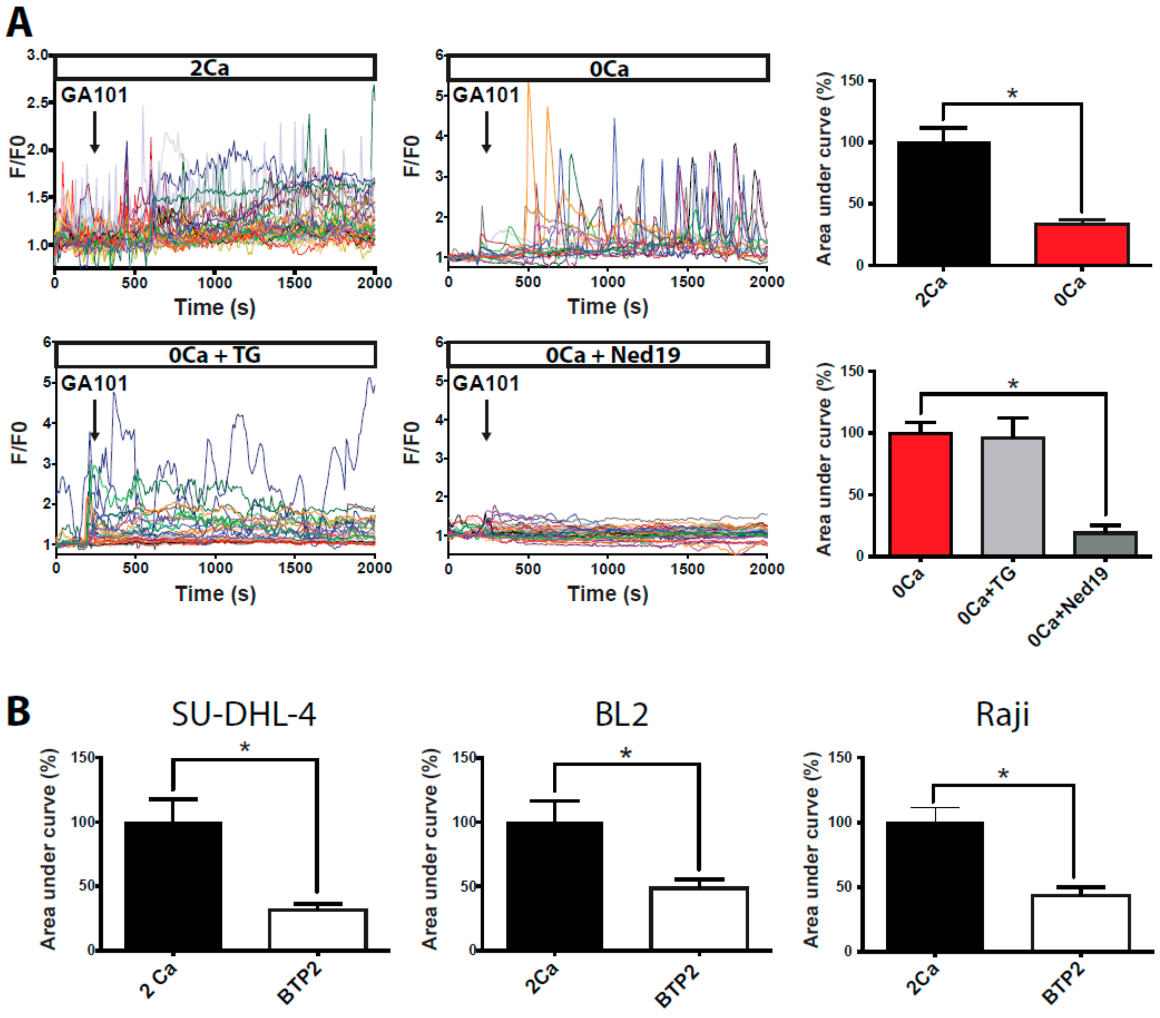

Figure S1. (**A**) Effect of GA101 on intracellular Ca^{2+} concentration in the Raji cell line. Ca^{2+} responses to GA101 (10 μg/mL) were measured using Fluo2-LR-AM Ca^{2+} dye and recorded by videomicroscopy (Zeiss LSM 510) using a 25× objective. Black arrows indicate GA101 addition. Each trace represents the response of one cell and data are representative of at least three independent experiments. Data were processed using OriginPro 7.5 (Origin Lab) or GraphPad prism. Cells were recorded in extracellular saline solution (HBSS) containing 2 mM Ca^{2+} (2Ca) or in Ca^{2+}-free HBSS (0Ca). Cells were preincubated with 100 nM thapsigargin (TG) for 45 min and recorded in Ca^{2+}-free HBSS (0Ca + TG), or with 10 μM Ned-19 for 1 h and recorded in Ca^{2+}-free HBSS (0Ca + Ned19). Histograms represent areas under curves (AUC) calculated, under various recording conditions, between the application time of GA101 and $t = 2000$ s; * $p < 0.05$. (**B**) Effect of SOCE inhibitor on GA101-induced Ca^{2+} response in SU-DHL-4, BL2, and Raji cells. Ca^{2+} response to GA101 (10 μg/mL) was measured as described in Figure 1. Cells were preincubated or not with BTP2 10 μM for 20 min and then recorded in HBSS

containing 2 mM Ca^{2+}. Areas under curves (AUC) were calculated under various recording conditions, between the application time of GA101 and $t = 2000$ s; * $p < 0.05$.

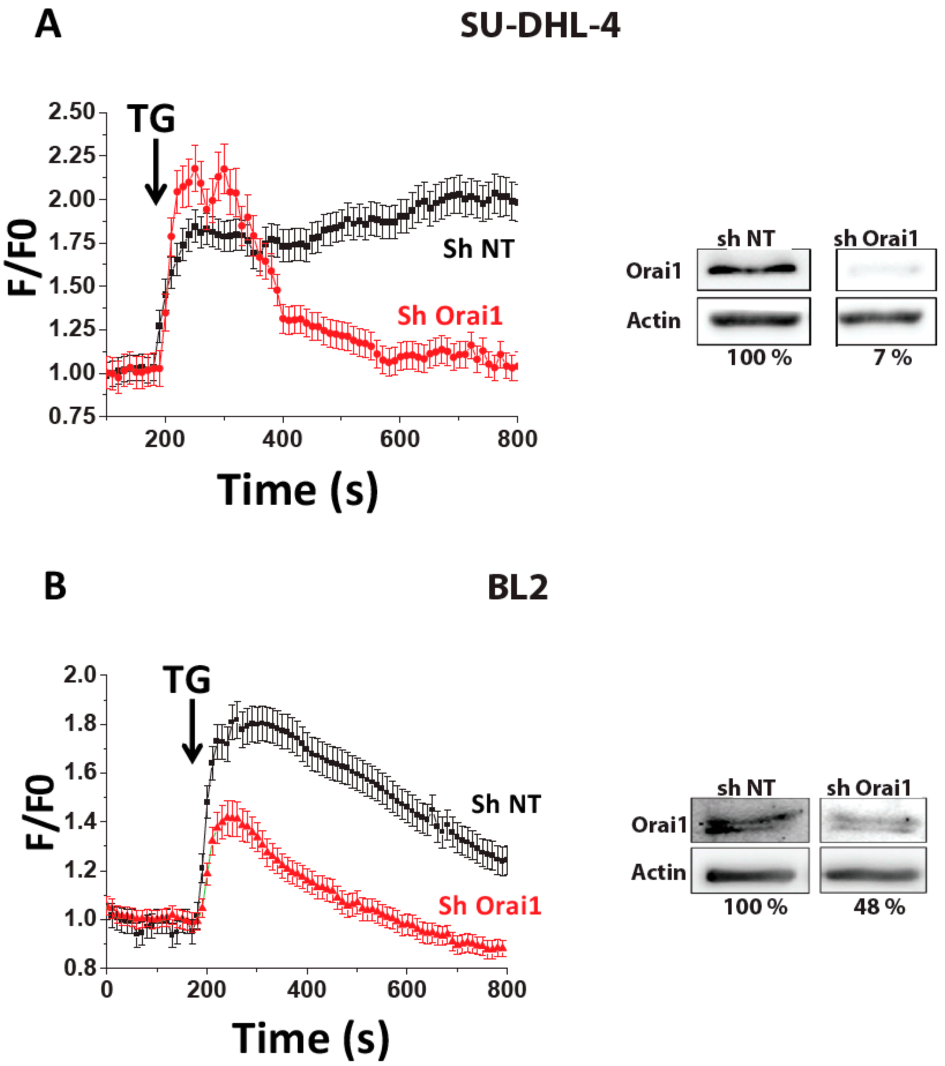

Figure S2. Validation of SU-DHL-4 (**A**) and BL2 (**B**) cell lines infected with nontargeting (sh NT) or Orai1-targeting shRNA (sh Orai1) lentiviruses. Ca^{2+} responses to TG (1 μM) were recorded in cells loaded with Fluo2-LR-AM in extracellular medium containing 2 mM Ca^{2+}. Recordings were performed using a conventional videomicroscopy set-up (Olympus IX-70 microscope, objective 40×). The data represent means ± SE of three independent experiments. Right panel: Orai1 expression level was evaluated by Western blot. β-actin was used as a loading control.

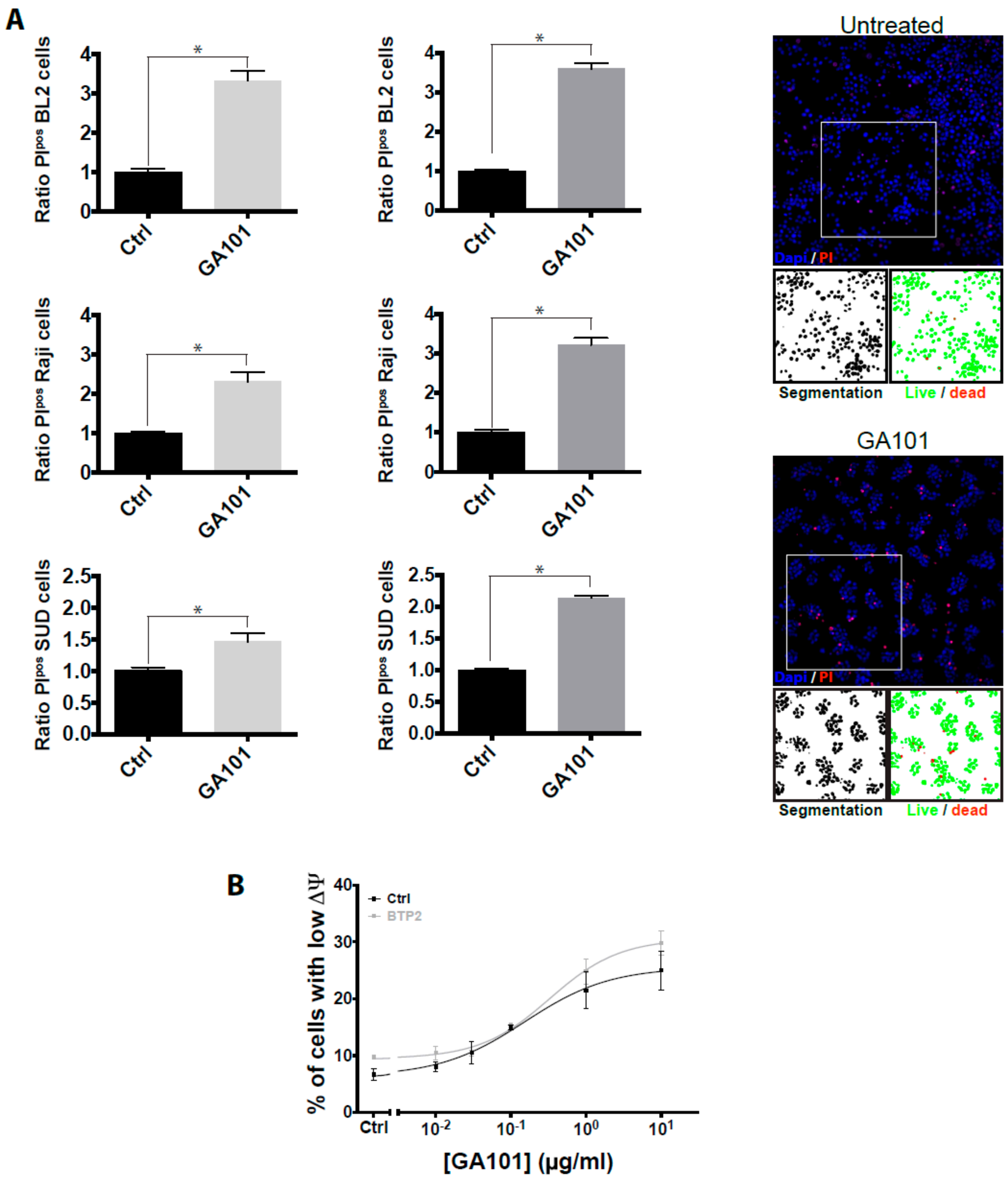

Figure S3. (**A**) Cell death induced by GA101 in various cell lines measured by microscopy (left panel). or flow cytometry (right panel). Cells were treated or not with GA101 (1 µg/mL) for 24 h. For determination of cell death by microscopy, cells were then labeled with propidium iodide (PI) (10 µg/mL) and Hoechst (1 µg/mL) for 30 min. Then, two fields were randomly imaged using a Leica DMI 8 epifluorescent microscope. Each condition was in duplicate and three independent experiments were performed. The percentage of PI-positive (dead cells)/Hoechst-positive cells (total cells) was determine by Image J software using an automatic macro. Briefly, a segmentation was performed based on Hoechst staining, and dead cells were defined as cells in which red signal (PI) was detected in segmented cells. For cell death measurement by flow cytometry, cells were labeled with PI for 30 min before analysis; * $p < 0.05$. (**B**) Involvement of SOCE in GA101-induced cell death in Raji cells. Cells were incubated with GA101 in the presence or absence of BTP2 (10 µM) for 24 h. Cell death was assessed by measuring the loss of mitochondrial membrane potential (ΔΨm), using TMRM as a fluorescent dye, and analyzed by flow cytometry.

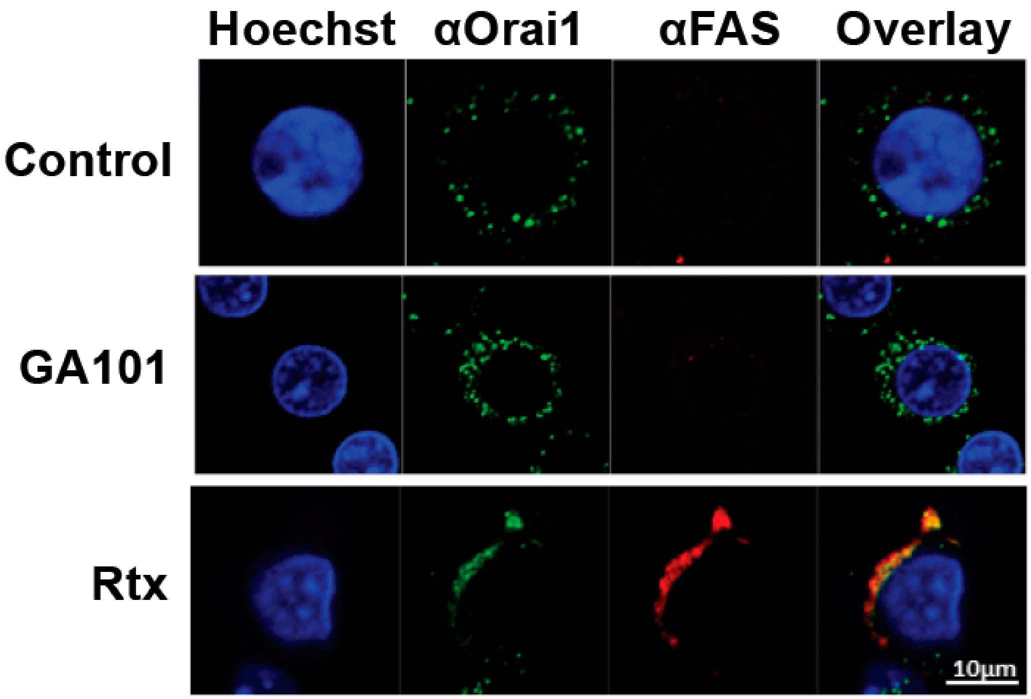

Figure S4. Representative images of immunostaining showing colocalization of Orai1 with CD95 in capping in SU-DHL-4 cells treated with RTX but not with GA101. Cells were incubated in the presence or not of RTX (10 µg/mL) or GA101 (10 µg/mL) at 37 °C for 15 min. Cells were fixed and stained with anti-CD95 revealed by donkey anti-mouse Ab coupled with Alexa-594 (red) and anti-Orai1 donkey anti-rabbit Ab coupled with Alexa-488 (green). Nuclei are depicted in blue. Images were acquired as described in Figure 4. Scale bar: 10 µm.

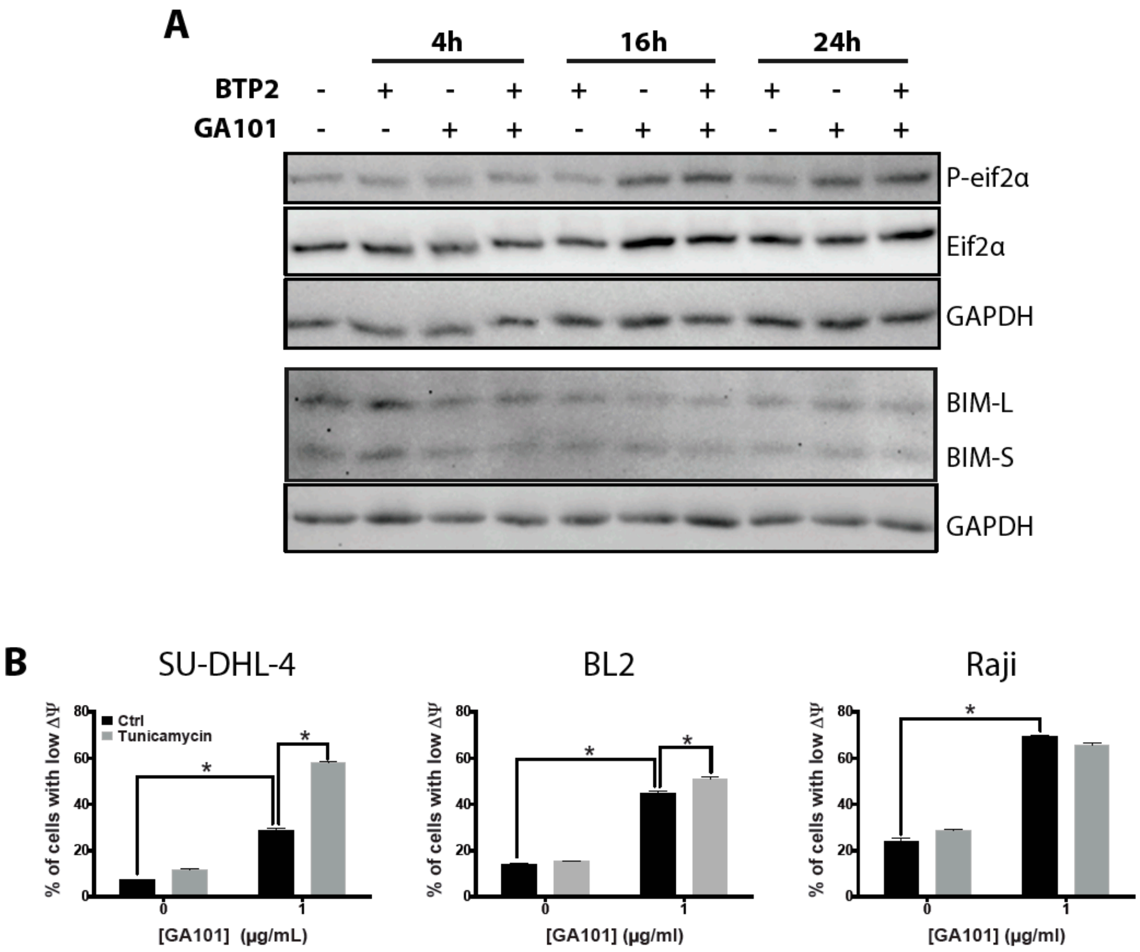

Figure S5. Involvement of ER stress in GA101-induced cell death. (**A**) Raji cells were treated with GA101 (1 µg/mL) in the presence of BTP2 (10 µM) for varying lengths of time. After lysis, P-eIF2*α*, eIF2*α*, and BIM expression levels were assessed by immunoblot analysis. GAPDH was used as a loading control. (**B**) Effect of the ER stress inducer, tunicamycin, on SU-DHL-4, BL2, and Raji cell death. Cells were incubated with GA101 (1 µg/mL) in the presence or absence of tunicamycin (10 ng/mL) for 24 h. Cell death was assessed by measuring the loss of mitochondrial membrane potential (Δψm), using TMRM as a fluorescent dye, and analyzed by flow cytometry; * $p < 0.05$.

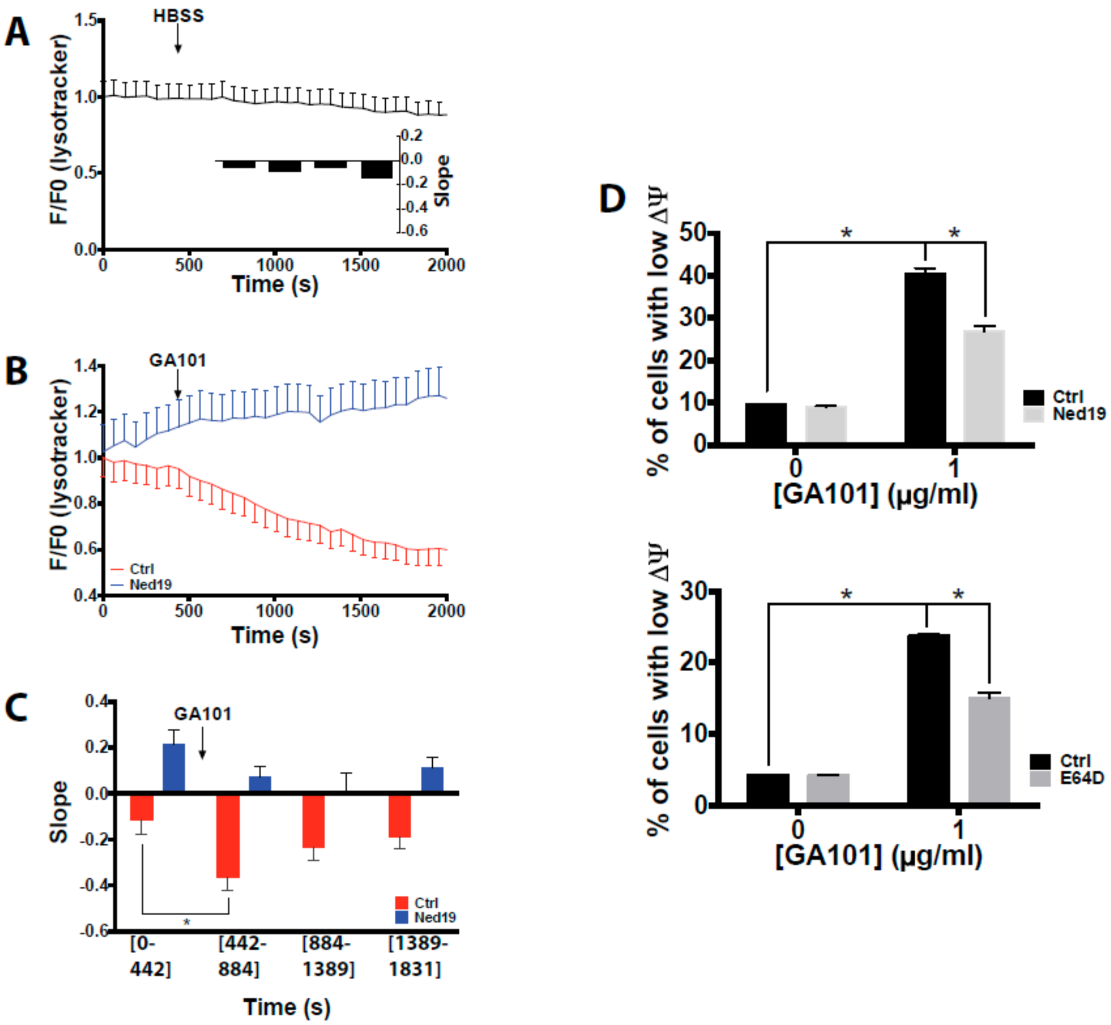

Figure S6. (**A**) Control recordings of lysotracker fluorescence. The fluorescence of lysotracker red DND-99 was recorded in Raji cells by videomicroscopy (Zeiss LSM 510) with a 63× objective. Black arrows indicate HBSS addition. (**B**) Effect of GA101 (10 µg/mL) on LMP in the Raji cell line. After pretreatment (in blue) or not (in red) with Ned-19 (10 µM) for 45 min, cells were recorded in HBSS containing 2 mM Ca^{2+}. Black arrow indicates GA101 addition. Each trace represents the mean ± SE of three independent experiments. (**C**) Quantification of the lysotracker fluorescence slope before and after the addition of GA101 in Raji cells after pretreatment or not with Ned-19. (**D**) Effect of LMP inhibition on GA101-induced cell death in Raji cells. Cells were incubated with GA101 in the presence or absence of Ned-19 of E64D for 24 h. Cell death was assessed by measuring the loss of mitochondrial membrane potential (ΔΨm), using TMRM as a fluorescent dye, and analyzed by flow cytometry; *$p < 0.05$.

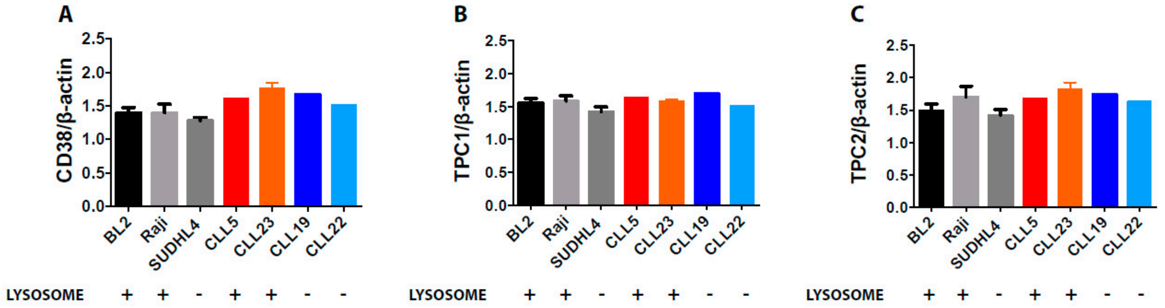

Figure S7. Messenger RNA expression level of CD38, TPC1, and TPC2 in NHL cell lines and B-CLL. Expression level of CD38 (**A**), TPC1 (**B**) and TPC2 (**C**) was determined by RT-qPCR on BL2, Raji, and SU-DHL-4 cell lines and in four CLLs. Messenger RNA expression of the various transcripts was normalized regarding β-actin expression. The involvement of lysosome in GA101-induced Ca^{2+} response is indicated by (+) or (−).

© 2019 by the authors. Licensee MDPI, Basel, Switzerland. This article is an open access article distributed under the terms and conditions of the Creative Commons Attribution (CC BY) license (http://creativecommons.org/licenses/by/4.0/).
